# Integrated Transcriptomic Analysis of *NOTCH1-* and *MYB*-Associated Immune Features in SACC

**DOI:** 10.3390/ijms27146498

**Published:** 2026-07-22

**Authors:** Guoliang Yang, Xudong Wang, Tian Ye, Tingyao Ma, Youmei Chen, Fang Nan, Lu Kong, Xiaohong Chen

**Affiliations:** 1 Department of Otolaryngology Head and Neck Surgery, Beijing Tongren Hospital, Capital Medical University, Beijing 100730, China; ygliang1016@mail.ccmu.edu.cn (G.Y.);; 2 Key Laboratory of Otolaryngology Head and Neck Surgery, Capital Medical University, Ministry of Education, DongJiaoMinXiang Street, DongCheng District, Beijing 100730, China; 3 School of Basic Medical Sciences, Capital Medical University, Beijing 100069, China; 4 Department of Biochemistry and Molecular Biology, Capital Medical University, No. 10 Xitoutiao, You An Men, Beijing 100069, China

**Keywords:** salivary adenoid cystic carcinoma, immune-desert, *MYB* fusion, *NOTCH1* mutation, B-cell receptor, antigen presentation

## Abstract

Salivary adenoid cystic carcinoma (SACC) is an immunologically cold malignancy with limited response to current immunotherapies. Integrated transcriptomic profiling of peripheral blood, primary tumors, lung metastases, and a two-donor single-cell dataset revealed compartment-specific expression signatures and, through computational inference, systemic immune dysregulation marked by hematopoietic suppression, T-cell exhaustion, compensatory myelopoiesis, and an immature B-cell expansion. To explore the transcriptional basis of this peripheral immune aberration, blood-derived RNA-seq was interrogated, identifying only 32 unique genes meeting |log2FC| > 1 and *q* < 0.05 among 34,999 transcripts; qPCR confirmed concordant upregulation of *IL33* and *CCL14*, providing directional rather than confirmatory support, suggesting peripheral immune molecular aberrations that still require validation through broader differential gene expression validation. Complementing this transcriptomic signature, detection of *MYB-NFIB* fusion transcripts matching tumor tissue in one patient’s blood suggested that tumor-derived signals may access the circulation, although cohort validation remains necessary. Extending these peripheral observations to tissue compartments, we applied expression stratification, correlation networks, ligand-receptor mapping, and a virtual gain-loss model to computationally predict regulatory associations involving *IL17RB/OLIG1/NOTCH1* in primary tumors and a *CD24/IL17RB/MYB/MYBL2/CXCL13/CXCR5* module in lung metastases. At the single-cell level, cluster 10 emerged as a cell-cycle-high tumor population with transcriptional overlap with proliferating immune progenitors, providing a potential cellular basis for tumor cell entry into the circulation. Collectively, these computational inferences generate testable hypotheses for multicompartment immune dysregulation in SACC, positioning *IL17RB* as a candidate molecule that warrants prospective validation in SACC-specific preclinical models.

## 1. Introduction

Salivary adenoid cystic carcinoma (SACC) represents a prototypical immune “cold” tumor with a sparsely infiltrated microenvironment and limited responsiveness to current immunotherapies [1,2]. Immune checkpoint inhibitors (ICIs) achieve response rates below 10% in this setting [3,4]. Defined by *MYB-NFIB* fusions [5], frequent *TP53/NOTCH1* mutations, low tumor mutational burden (TMB < 2 mut/Mb) and microsatellite stability [3,6], SACC follows an indolent yet ultimately lethal course: over 50% of patients develop metastatic lung disease within a decade [7], collapsing five-year survival from ~70% to under 20% [8]. Peripheral-blood transcriptomic analysis in the current cohort identified exploratory B-cell receptor (BCR)-associated and erythroid-lineage expression changes. Given the established relevance of BCR signaling and immune-state diversity in cancer [9,10], these observations motivate investigation of systemic immune and hematopoietic dysregulation but do not establish bone-marrow dysfunction, limited by ethical constraints that preclude investigation in SACC patients.

*MYB-NFIB* fusions mimic a C-terminally truncated virus *v-MYB*, driving constitutive transcriptional activation [11]. *MYB* and its homologs (*MYBL1*, *MYBL2*) play distinct roles in hematopoiesis. *MYB* is a critical transcription factor in hematopoietic stem cells (HSCs) and multilineage progenitors [12] and is involved in invariant natural killer T cell (iNKT) differentiation via CD1d/SLAMF/SAP signaling [13,14]. In contrast, *MYBL1-* predominantly expressed in tonsillar, thymic, and splenic B cells—regulates proliferation and differentiation of mature B lymphocytes, while *MYBL2* enhances T/NK cell cytotoxicity [15]. Notably, heterozygous *Mybl2*-deficient mice develop age-dependent bone marrow disorders. Tumor-expressed *NOTCH1* also exerts specific immune-modulatory effects, for example, gender-specific regulation of dendritic and T-cell-mediated anti-tumor responses in hepatocellular carcinoma [16]. Although its role in T-lineage commitment (particularly γδT-cell development) is well-established, a broader understanding of how *NOTCH1* influences immune ontogeny remains elusive [17,18]. Importantly, emerging evidence reveals that tumors systemically reprogram bone marrow-derived immune cells via circulating immunosuppressive populations [19]. Whether SACC-associated driver alterations are linked to systemic progenitor- or immune-cell changes remains unknown.

The Human Cell Mapping Initiative has defined 44 to 51 T-cell subtypes through transcriptomic profiling of primary lymphoid organs (bone marrow, thymus), secondary lymphoid organs (lymph nodes, spleen), and peripheral blood [20]. Additionally, spatial transcriptomics now allows multi-scale immune decoding of immune architecture, mapping interactions from local tumor microenvironments to coordinated immune activity across tissues [21,22]. This powerful approach has revealed distributions of epidermal/subdermal dendritic cells, tissue-resident memory T-cells (TRM), and macrophages in non-lymphoid organs, deepening insights into disease mechanisms [23,24,25].

In this study, the *MYB-NFIB* fusion gene, consistent with that found in tumor tissue, was detected for the first time in the peripheral blood of a patient with SACC, using a combined strategy of Sanger sequencing and nested PCR (Supplemental Figure S1). As only one patient was tested and no independent negative clinical-control series was available, this finding is treated as a proof-of-principle observation. By integrating single-cell and bulk RNA-seq data from SACC tumors, metastases, and blood, together with a public single-cell atlas [26], the immune microenvironment and regulatory networks in SACC were systematically characterized through enrichment-score-based methods. Through binarization and stratification of the data, gene “knockdown” and “overexpression” were computationally simulated to predict upstream regulators and downstream effectors of the regulatory genes. Key pathways are based on computational predictions and await confirmation through large-scale data validation and functional experiments.

## 2. Results

### 2.1. Distinct Immune Profiles in Blood, Primary Tumors, and Lung Metastases

**Figure 1:**
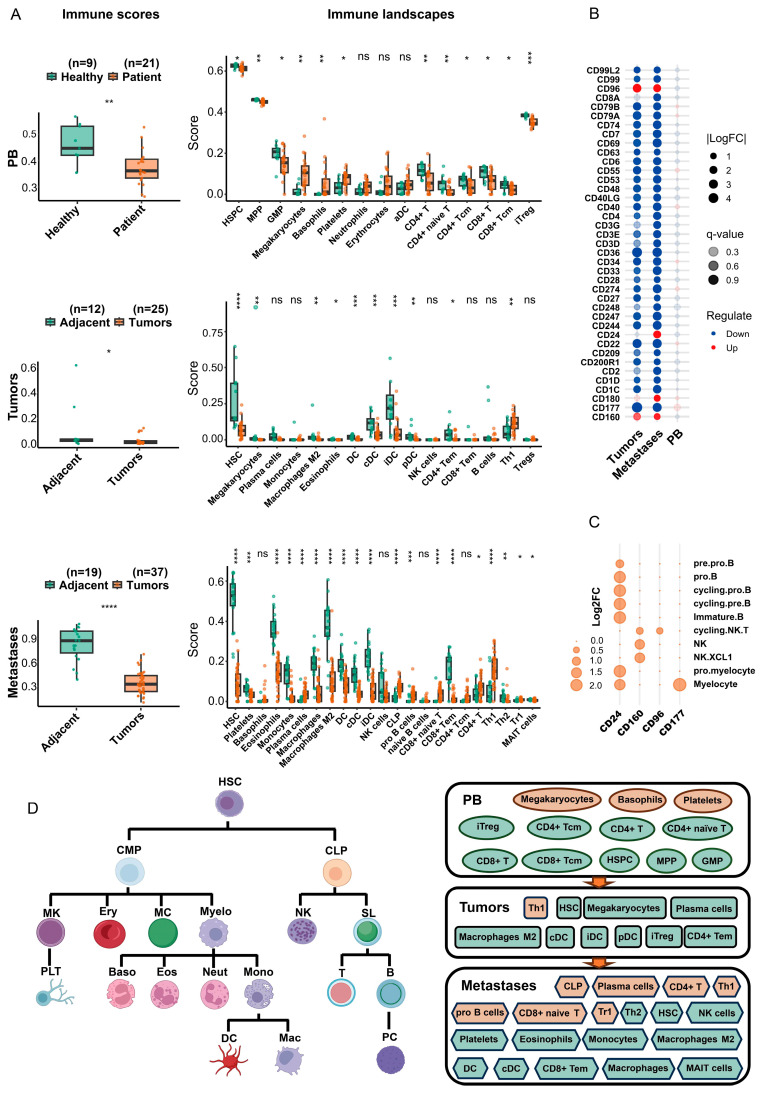
Exploratory immune profiles and LDAs expression in PB, primary tumors, and lung metastases of SACC patients. (**A**) Immune scores and immune cell composition across different compartments. Top: PB of SACC patients (n = 21) vs. healthy controls (n = 9); middle: primary tumors (n = 25) vs. adjacent non-tumor tissues (n = 12); bottom: lung metastases (n = 37) vs. adjacent normal tissues (n = 19). Light green indicates healthy individuals or adjacent non-tumor tissues, and light red indicates SACC patients or tumor tissues. Mann-Whitney U test, *p* > 0.05 (ns), *p* < 0.05 (*), *p* < 0.01 (**), *p* < 0.001 (***), *p* < 0.0001 (****). (**B**) Differential expression patterns of LDAs in PB, primary tumors, and lung metastases of SACC patients. The size of the bubbles represents the logFC values. (**C**) Compartment-specific upregulation of LDAs shows unique immune cell signatures in PB, primary tumors, and metastatic tissues. The single-cell data were derived from the public dataset ABC. (**D**) Schematic illustrating hematopoietic differentiation (Left) and multi-compartment immune cell alterations (Right). Light red and green indicate upregulation and downregulation, respectively.

SACC patients demonstrated systemic immunosuppression, with significantly reduced immune scores across peripheral blood (PB), primary tumors, and pulmonary metastases, with distinct inferred immune patterns in each compartment (Figure 1A).

PB deconvolution suggested lymphoid depletion and compensatory myelopoiesis compared to healthy controls. Lower inferred populations included hematopoietic stem/progenitor cells (HSPCs), multipotent progenitors (MPPs), granulocyte-macrophage progenitors (GMPs), CD4^+^ naïve T cells, induced regulatory T cells (iTregs), conventional CD4^+^ and CD8^+^ T cells, and central memory T cells (Tcm). In contrast, megakaryocytes, Basophils, and platelets were elevated, alongside increasing trends in monocytes, erythrocytes, and neutrophils (Figure 1A, right, top).

Primary-tumor deconvolution suggested selective Th1 enrichment yet widespread suppression of effector lineages. Significantly reduced components encompassed HSCs, megakaryocytes, plasma cells, M2 macrophages, conventional and immature dendritic cells (cDCs, pDCs and iDCs), and CD4^+^ effector memory T cells (Tem) were all significantly reduced (Figure 1A, right, middle).

Lung-metastasis deconvolution suggested a paradoxically ‘hot’ microenvironment with lymphoid activation and myeloid suppression, and expanded populations included plasma cells, early lymphoid progenitors (CLPs and pro-B cells), and multiple T cell subsets (CD8^+^ naïve, CD4^+^, Th1, and Tr1) (Figure 1A, right, bottom). However, persistent deficiencies were observed in crucial cytotoxic effectors, including T cells (CD8^+^ Tem, MAIT, Th2), innate immune cells (DCs, cDCs, macrophages, M2 macrophages, monocytes, eosinophils, NK cells), and hematopoietic/prothrombotic elements (HSCs, platelets). This impairment signifies ongoing immune evasion despite local immune activation.

Leukocyte differentiation antigens (LDAs) showed compartment-specific expression patterns: *CD24* and *CD180* were uniquely upregulated in lung metastases; *CD177* was elevated in blood but down in both primary tumors and metastases; and *CD96* and *CD160* were elevated in both tumor compartments (Figure 1B). A public single-cell transcriptional atlas of blood cells (ABC) (Supplemental Figure S2A) indicated *CD24*’s expression in early B-lineage, particularly in pre-B cells, and in myeloid progenitors, *CD180* in antigen-presenting cells (e.g., B cells and DCs), and *CD96/CD160* in proliferating NK and T cells, while *CD177* marks myeloid lineages (Figure 1C). These reference patterns provide cell-type context for, but do not functionally validate, the compartment-level transcriptomic associations summarized in Figure 1D.

### 2.2. Peripheral-Blood Transcriptomic Changes in SACC

**Figure 2:**
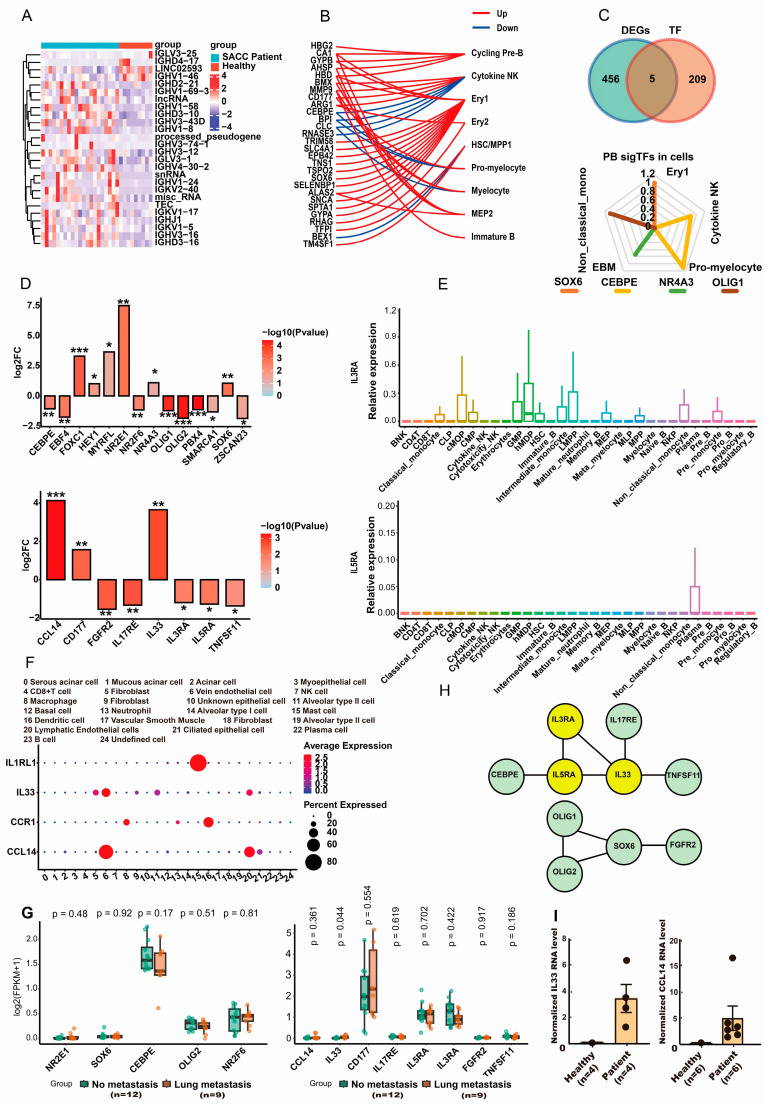
Functional Profiling of peripheral-blood transcriptomic candidates in SACC. (**A**) Heatmap depicting the expression patterns of 26 selected immunoglobulin-related or novel transcripts in the peripheral blood of healthy donors (HDs) versus SACC patients. (**B**) Immune cell type association of peripheral blood nominal DEGs. Nominal DEGs identified from SACC patients compared to HDs were projected onto immune cell lineages using the ABC database. Red and blue colors indicate up- and down-regulation, respectively. (**C**) Analysis of nominally significant TFs. Venn diagram illustrating the overlap of nominally significant TFs (upper). Radar chart visualizing the annotated immune cell types for these TFs (bottom), based on the ABC single-cell dataset. (**D**) Profile of nominally significant TFs (upper) and cytokines (bottom), shown by log_2_FC values. *p* < 0.05 (*), *p* < 0.01 (**), *p* < 0.001 (***). (**E**) Expression patterns of *IL3RA* and *IL5RA* across immune cell populations, as annotated in the ABC database. (**F**) Expression of *CCL14-CCR1* and *IL33-IL1R1* ligand-receptor pairs across 25 single-cell clusters from two SACC patients. (**G**) Within-cohort comparison by metastatic status; subgroup sizes are shown in the panel. (**H**) Protein-protein interaction network constructed using the STRING database. (**I**) Validation by qPCR on blood samples confirmed elevated expression of *IL33* and *CCL14* in SACC patients (n = 6).

Blood transcriptome nominally significant candidate differentially expressed genes (DEGs) were defined using the thresholds |log_2_FC| > 1 with *p* < 0.05. 26 novel differentially expressed transcripts in PB, including 15 *IGH* genes (10V, 4D, 1J), 5 light chain genes (3 IGκ, 2 IGλ), and 6 non-antibody genes (4 non-coding RNAs, 1 pseudogene, 1 unconfirmed transcript) were classified using immunoglobulin gene-segment nomenclature (IMGT^®^) [27]. Except for *IGHV1-46, IGHD4-17, IGLV3-25,* and *LINC02593,* all showed upregulated (Figure 2A, Supplemental Table S1). These BCR anomalies reflect stage-specific developmental failures: altered D-segment usage during the pro-B stage (*IGHD4-17* suppression with *IGHD3-10* dominance), defective VDJ recombination or pre-BCR checkpoint failure in the pre-B stage (*IGHV3-74-1* overexpression with low *IGHV1-46*), and immature B cell maturation arrest (low *IGLV3-25* with skewed κ-chain dominance via *IGKV1-5*) [28].

Mapping the nominal candidate DEGs set to the healthy ABC reference atlas suggested enrichment of cycling pre-B-cell, immature B-cell, and erythroid-associated programs (Figure 2B). The analysis further highlighted lineage-associated transcription factors (TFs), with upregulated expression of *SOX6* (erythroid-restricted) and *NR4A3* (immature B cells), and downregulated expression of *OLIG1* (non-classical monocytes) and *CEBPE* (myeloid precursors) (Figure 2C,D; Supplementary Table S2).

Cytokine profiling in SACC patients showed dual dysregulation: elevated alarmin *IL-33* and chemokine *CCL14*, along with reduced expression of immunoregulatory receptors *IL-5RA, IL-3RA, IL-17RE, FGFR2,* and *TNFSF11* (Figure 2D, bottom). ABC datasets indicate that *IL3RA* is broadly expressed across hematopoietic lineages, and *IL5RA* mainly in plasma cells (Figure 2E). Single-cell ligand-receptor mapping generated candidate *CCL14-CCR1* and *IL33-IL1R1* interactions involving endothelial, dendritic-cell, macrophage, and mast-cell states (Figure 2F). Among all dysregulated factors, only *IL-33* levels specifically correlated with lung metastasis (Figure 2G), and STRING analysis predicted an interaction network between IL33 and IL3RA, IL5RA, IL17RE, and TNFSF11. This hypothesis is supported by predicted protein-protein interaction networks rather than direct biochemical evidence of protein binding (Figure 2H). Blood qPCR validated the upregulation of *IL33* and *CCL14* in SACC patients compared to controls (Figure 2I). Furthermore, pan-cancer analysis revealed that elevated *IL33* expression is significantly associated with poor overall survival across multiple cancer types (Supplemental Figure S2B,C).

### 2.3. Transcriptomic Cytokine and Monocyte/DC-Associated Patterns in Primary ACC

**Figure 3:**
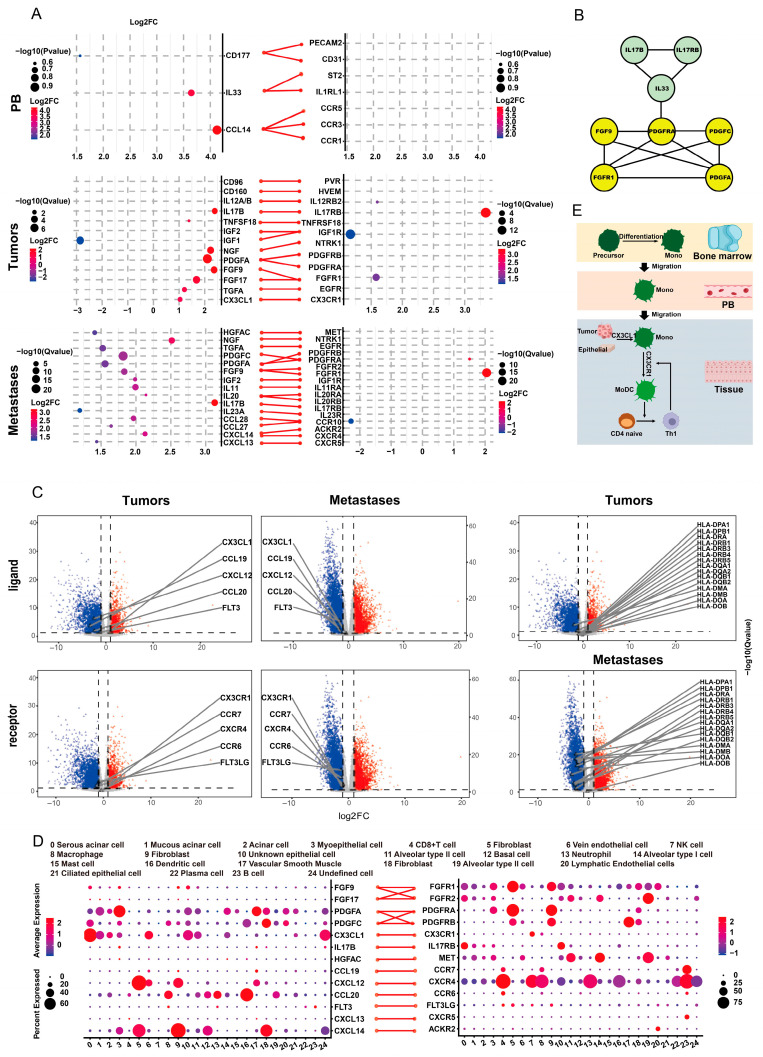
Characterization of cytokine network alterations in SACC. (**A**) Differential receptor-ligand expression in peripheral blood (21 patients with SACC vs. 9 healthy controls), primary tissue (25 tumors vs. 12 adjacent tissues), and lung tissue (37 metastases vs. 19 adjacent normal tissues); color represents log_2_FC and dot size represents −log10(*p/q*). (**B**) Predicted functional interactome among the altered cytokines, mapped using the STRING database. (**C**) Volcano plot highlighting key DC maturation and homing chemokines and ligands. (**D**) Hypothesis-generating schematic of monocyte differentiation into monocyte-derived dendritic cells (moDCs). (**E**) Single-cell resolution maps from two SACC patients, annotating the specific cell types expressing altered receptor-ligand pairs.

Cytokine profiles across peripheral blood, primary tumors and lung metastases were compared to identify, via computational prediction, candidate correlates of lymphopenia. The differentially misregulated cytokines and their ligands are detailed in Figure 3A. Three cytokine networks were highlighted: *IL17B-IL17RB* (in both sites), *FGF9/17-FGFR1* (primary tumors), and *PDGFA/C-PDGFRA/HGFAC-MET* (metastases) (Figure 3A). In the two-donor single-cell dataset, tumor cells expressing *IL-17RB* interacted with stromal or cluster 24-derived *IL-17B* (Figure 3D); this communication is absent in circulating immune cells (Figure 3A, bottom). STRING analysis linked IL33, PDGFRA, and IL17B-IL17RB as a candidate association network (Figure 3B).

Primary tumors exhibited elevated levels of *CX3CL1*, a cytokine important for DC chemotaxis, coinciding with increased inferred infiltration of DC precursors. Notably, *CX3CL1* (*CX3CR1* ligand) expression was unchanged in primary tumors but suppressed in metastases (Figure 3C, left and middle). Curiously, key DC maturation/homing chemokines (*CCL19-CCR7, CXCL12-CXCR4, CCL20-CCR6,* and *FLT3-FLT3LG)* and major histocompatibility complex (MHC) II were downregulated in both tumor contexts (Figure 3C). Collectively, these patterns imply that tumor-infiltrating DCs may be related to monocyte precursors rather than classical myeloid progenitors.

Single-cell expression maps further indicated that *CX3CL1* was detected mainly in tumor cells, vascular endothelial cells, and cluster 24 cells within the primary lesions, whereas *CX3CR1* was predominantly expressed on NK cells and monocytes (Figure 3D). These patterns suggest a model in which the *CX3CL1-CX3CR1* axis may be associated with recruitment or retention of *CX3CR1^+^* immune cells (e.g., NK cells, monocytes), as well as the subsequent differentiation into monocyte-derived dendritic cell states (Figure 3E).

### 2.4. Transcriptomic Characterization of Dendritic Cell Progenitor Differentiation

**Figure 4:**
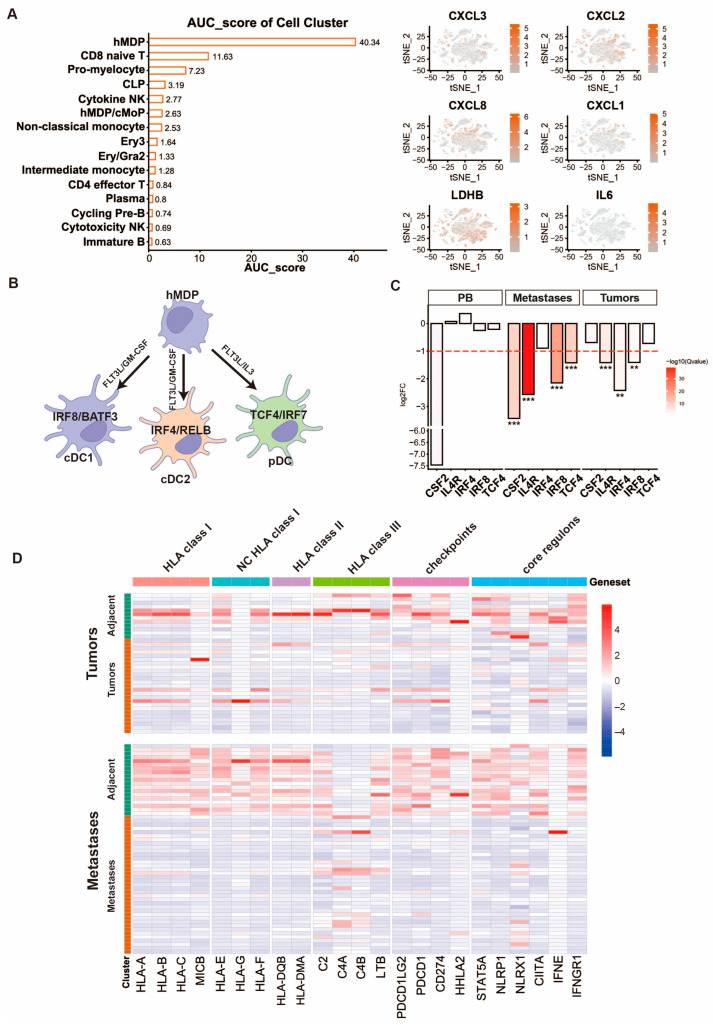
Transcriptomic features associated with hMDP-like and dendritic-cell states. (**A**) Single-cell data annotation identified cluster 24 as hMDP-like cells (51,128 cells; six specimens; two unmatched donors). Left: AUC score (sum of AUC scores for cluster 24-specific marker genes; data from ABC). Right: t-SNE plot displaying cytokines common to hMDP and cluster 24. (**B**) Schematic of key drivers regulating hMDP differentiation into DC subsets. (**C**) Differential expression of selected DC-lineage factors in peripheral blood (21 SACC vs. 9 healthy), primary tissue (25 tumors vs. 12 adjacent tissues), and lung tissue (37 metastases vs. 19 adjacent normal tissues). *q* < 0.01 (**), *q* < 0.001 (***). (**D**) Changes in antigen-presenting molecules across different sites. Genes with |log_2_FC| > 1 and *q* < 0.05 are shown.

Reanalysis of the two-donor single-cell data identified cluster 24 (exclusive to primary tumors) as human myeloid dendritic cell progenitor-like (hMDPs-like) cells based on transcriptional similarity (Figure 4A; Supplemental Figure S3 and Table S3) [29]. Concurrent increases in blood monocyte frequency and tissue-resident Th1 cells suggest an alternative function: CX3CL1 derived from hMDP-like cells was associated with the recruitment of circulating CX3CR1^+^ monocytes to tumor sites, where they could differentiate into moDCs and potentially support Th1 cell responses through antigen presentation (Figure 3E).

Despite elevated iDC scores in metastatic sites (Figure 1A), the absence of essential cytokines was associated with impaired differentiation into functional cDCs or pDCs (Figure 4B). Hematopoietic factor analysis showed significant *CSF2* (GM-CSF) downregulation in metastases (trend in PB), and *IL4R* reduction in both tumor sites. Transcriptional profiling further suggested compartment-specific alterations in DC lineage regulators: *IRF8* (cDC1) was reduced in both tumor sites; *IRF4* (cDC2) exclusively in primary tumors; and *TCF4* (pDCs) selectively in metastases. These changes were not observed in PB (Figure 4C).

This coincides with reduced expression of antigen-presenting machinery, including classical HLA class I (*HLA-A/B/C, MICB*), non-classical HLA class I (*HLA-E/G/F*), HLA class II (*HLA-DQB1/DMA*), HLA class III (*C2, C4A/B, LTB*), immune checkpoint (*PDCD1LG2, PDCD1, CD274, HHLA2*), core regulons (*STAT5A, NLRP1, NLRX1, CIITA, IFNE, IFNGR1*). Notably, the lung-metastasis cohort showed broader lower expression across *HLA classes I/II* compared to primary tumors (Figure 4D).

### 2.5. NOTCH1 Is Associated with an Immune-Desert Phenotype in Primary Tumors

**Figure 5:**
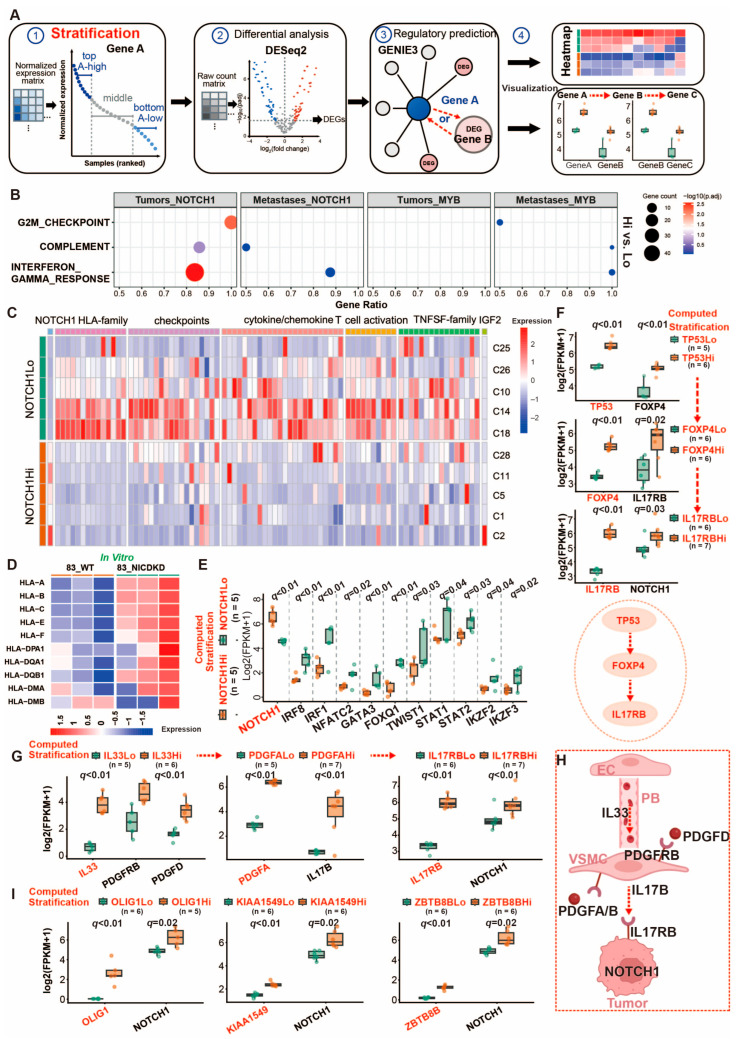
Computationally inferred *NOTCH1*-associated networks in primary SACC. (**A**) Schematic of the stratified-expression workflow, including expression grouping, differential-expression analysis, regulatory-network prediction and visualization. Red dashed lines indicate the direction of the predicted regulatory relationships. (**B**) Hallmark pathway (MSigDB) enrichment analysis of DEGs from *NOTCH1/MYB* stratification (high vs. low) in primary tumors and lung metastases (DEGs defined by DESeq2). (**C**) Genes involved in antigen presentation, interferon signaling, and lymphocyte development that were negatively associated with the *NOTCH1*-high state. (**D**) *HLA* transcript responses after NICD1 knockdown in SACC-83 cells. (**E**) Higher *NOTCH1* expression was negatively associated with the expression of genes involved in antigen presentation (*IRF8*, *IRF1*), immunomodulation (*GATA3*, *FOXQ1*), interferon response (*STAT1/2*), and lymphocyte differentiation (*IKZF2/3*). (**F**) Computationally inferred associations among *TP53*, *FOXP4*, *IL17RB*, and *NOTCH1* in primary tumor. (**G**) Predicted associations in the *IL33-PDGFRB/PDGFD-PDGFA/B-IL17B-IL17RB-NOTCH1* network. (**H**) Schematic: endothelial-smooth-muscle-cell paracrine signaling. (**I**) Transcription factors computationally associated with *NOTCH1* expression.

*MYB* and *NOTCH1* overexpression define molecular features of SACC [11]. To investigate their potential role in blocking DC differentiation, an expression-based stratification strategy was used to examine associations between *MYB* and *NOTCH1* expression and immune-related transcripts (Figure 5A). Compared to the *MYB*-high group, the *NOTCH1*-high group exhibited significantly lower expression of immune-related genes (log_2_FC > 1, *q* < 0.05), enriching for IFN-γ response, complement inactivation, and G2M checkpoint (Figure 5B). Moreover, *NOTCH1* -driven transcriptional effects diverged sharply between primary tumors and lung metastases (Supplemental Table S4).

In primary sites, genes expressed at lower levels in the *NOTCH1*-high stratum fell into five categories: (1) HLA-family, (2) immune checkpoints (co-stimulatory/inhibitory), (3) cytokine/chemokine signaling, (4) T-cell activation markers, and (5) tumor necrosis factor superfamily (TNFSF) members involved in inflammatory regulation (Figure 5C). In vitro experiments demonstrated that NICD1 knockdown (resulting in *NOTCH1* downregulation, *q* = 0.00) in SACC-83 cells restored HLA molecule expression, supporting a role for the NOTCH1 pathway (Figure 5D). In contrast, NICD1 knockdown in the SACC-LM cell line (no significant change in *NOTCH1* expression, *q* = 0.44) instead led to downregulation of HLA molecules. *IGF2* was the only highlighted growth factor with higher expression, inversely correlated with HLA class II expression (Figure 5B). In this analysis, *NOTCH1* activation was associated with lower expression of genes involved in antigen presentation machinery (*IRF8*, *IRF1*, *STAT1*, *NFATC2*), immunomodulators (*GATA3*, *FOXQ1*, *TWIST1*), interferon response genes (*STAT1/2*), and lymphocyte differentiation factors (*IKZF2/3*) (Figure 5E), implying *NOTCH1*’s role in constraining antigen presentation, interferon signaling, and lymphocyte development.

Expression-stratification and network analyses nominated *IL17RB* as a putative upstream correlate of *NOTCH1* expression in tumor cells. Together, these analyses generated a computationally inferred *TP53*-*FOXP4*-*IL17RB*-*NOTCH1* model as a candidate contributor to pathogenic NOTCH1 overexpression in primary ACC (Figure 5F).

Given the high frequency of *PDGFRA* mutations in SACC fibroblasts [30], candidate upstream relationships involving PDGF-family members were also examined. Based on computational analysis and single-cell mapping, *PDGFA* and *PDGFB* expression in vascular smooth muscle cells was associated with *IL17B*, while endothelial-cell *IL33* was associated with *PDGFRB* and *PDGFD* expression. These results generated a candidate *IL33-PDGFRB/PDGFD-PDGFA/B-IL17B-IL17RB-NOTCH1* paracrine network (Figure 5G,H). Furthermore, computational analysis identified three transcription factors, *OLIG1*, *KIAA1549* and *ZBTB8B* as putative positive regulators of *NOTCH1* expression, with *OLIG1* predicted to be regulated exclusively by *IL17RB* within this network (Figure 5I).

### 2.6. Transcriptomic Associations Involving NOTCH1 and the CXCL13-CXCR5 Axis in Lung Metastasis

**Figure 6:**
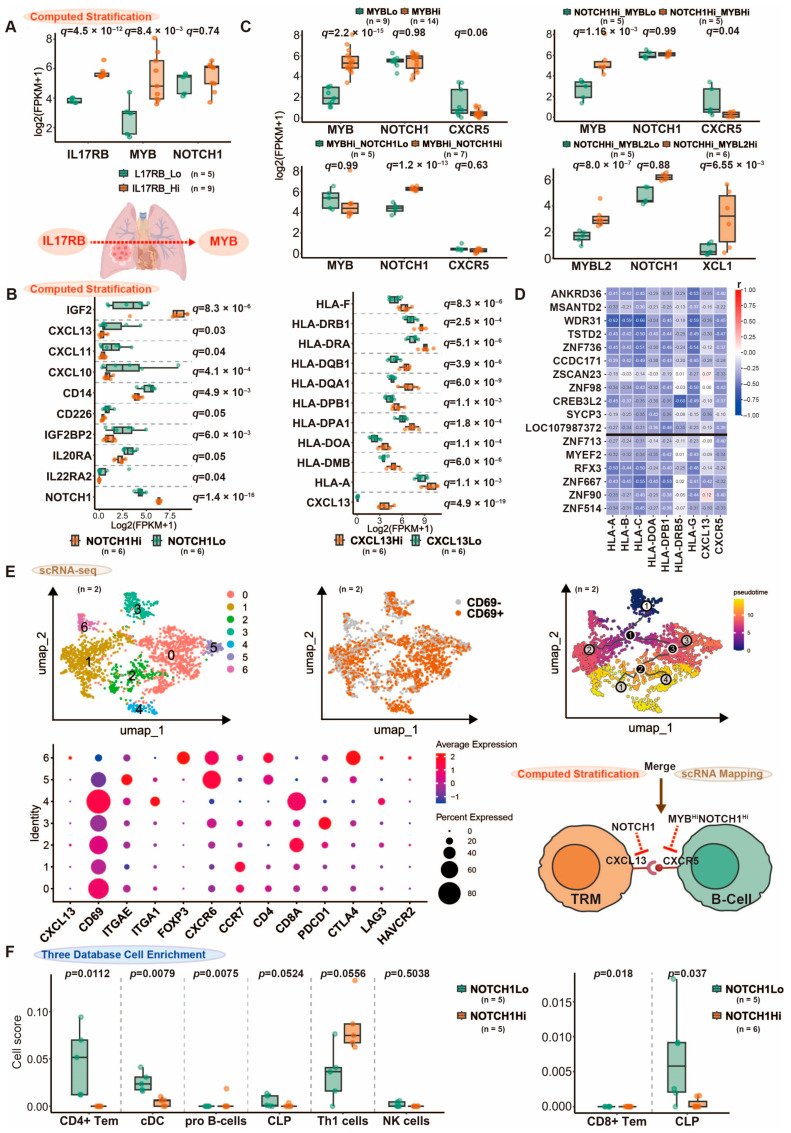
Transcriptomic associations involving the *CXCL13-CXCR5* axis in lung metastases. (**A**) Computationally predicted association between *IL17RB* and *MYB* expression. (**B**) Genes differentially expressed between *NOTCH1* strata and associations between *CXCL13* and *HLA* class I/II transcripts; *q*-values from DESeq2. (**C**) Computationally predicted relationships of *MYB/MYBL2* and *NOTCH1* with *CXCR5* and *XCL1* in metastatic tumors. (**D**) Negative correlation screening Identifies 17 genes (6 TFs) associated with lower HLA and T-cell-activation-marker expression. (**E**) CD8^+^ T cell (cluster 4) subset analysis: transcriptionally distinct TRM populations, a *CXCL13*-expressing Treg TRM, and a putative differentiation trajectory from activation to suppression (UMP and bubble plot). Schematic illustrating the proposed interactions between TRM-primed CD8^+^ T cell and B cell. (**F**) *NOTCH1*- associated immune cell patterns in primary (left) and metastatic lung tissues (right). Red denotes computational predictions, brown-yellow denotes single-cell observations, and green denotes experimental observations, where applicable. Sample sizes are indicated in the respective panels.

At metastatic sites, *IL17RB* was computationally predicted as an upstream candidate associated with *MYB* rather than a direct activator of *NOTCH1* (Figure 6A) This association was considered in the context of the previously reported MYB-related noncanonical NOTCH1 pathway [30]. Stratification analysis further indicated that high *NOTCH1* expression was not directly associated with lower expression of *HLA* genes or T-cell activation markers. Instead, high *NOTCH1* expression was associated with lower *IL22RA2*, *IL20RA*, *IGF2BP2*, *CD226*, *CD14*, *CXCL10*, *CXCL11*, and *CXCL13* with higher *IGF2* expression, a known repressor of *HLA* class II molecules (Figure 6B). Intriguingly, *CXCL13*, which is highly expressed in metastatic lesions, was associated with higher *HLA* class I and II expression (Figure 6B). These findings raise the possibility that higher *NOTCH1* expression may be associated with altered DC-related function, potentially through lower *CXCL13* expression and/or an *IGF2*-associated reduction in HLA class II expression. To further dissect this axis, metastatic samples were stratified by *MYB/MYBL2* and *NOTCH1* expression: (1) *MYB*-high vs. *MYB*-low, (2) *MYB*-high/*NOTCH1*-high vs. *MYB*-low/*NOTCH1*-high, and (3) *MYB*-high/*NOTCH1*-high vs. *MYB*-high/*NOTCH1*-low. *CXCR5* was approximately 20-fold lower in the *MYB*-high/*NOTCH1*-high versus *MYB*-low/*NOTCH1*-high comparison, and *XCL1*, a key chemokine required for cDC1 recruitment via *XCR1*, was approximately 20-fold lower in the *MYBL2*-high/*NOTCH1*-high versus *MYBL2-low/NOTCH1*-high comparison (Figure 6C). Negative correlation screening identified 17 genes associated with lower *HLA* and T-cell-activation-markers expression (Figure 6D). The majority were also associated with lower *CXCR5* or *CXCL13* expression, raising the possibility that disruption of the *CXCL13-CXCR5* axis as a candidate mechanism underlying antigen presentation failure. Core transcriptional repressors driving this network included *ZNF713*, *MYEF2*, *RFX3*, *ZNF667*, *ZNF98*, and *ZNF514* (Figure 6D).

Single-cell analysis localized *CXCL13* predominantly to CD8^+^ T cells and *CXCR5* expression confined to B cells (Figure 3D), aligning with inferred enrichment of CLP, pro-B, and naïve B cells in metastases (Figure 1A). It suggested that *CXCL13-CXCR5* axis may be associated with B-cell-mediated antigen presentation at metastatic sites. Subset analysis of CD8^+^ T cells (cluster 4) revealed: subsets 0-5 universally expressed *CD69*; subset 4 exclusively expressed *ITGA1* (CD49A); subset 5 uniquely expressed *ITGAE* (CD103); subset 6 specifically expressed *FOXP3* and *CTLA4*; subsets 3,5,6 co-expressed *CXCR6* and *PDCD1*; and subset 1 expressed *CCR7.* These expression patterns are consistent with all six subsets representing tissue-resident memory T cell (TRM) populations, encompassing CD8^+^, CD4^+^, and Treg TRM subtypes, suggesting the predominant TRM within metastases [31]. Notably, pseudotemporal trajectory analysis (monocle3) indicated a differentiation path from subset 3→1 and 1→0→2, suggesting a transition from activation toward suppression within metastases. Treg TRM (subset 6) emerged as the predominant *CXCL13*-expressing cell subset. The schematic figure illustrates the hypothesis integrating computational analysis and single-cell mapping. (Figure 6E, Supplemental Table S5).

Cell-type deconvolution further showed context-specific associations between *NOTCH1* expression and inferred immunosuppression in the tumor microenvironment. In primary tumors, higher *NOTCH1* expression correlated with lower CD4^+^Tem and cDCs scores but a higher pro-B-cell score, while in metastases, it was associated with lower CD8^+^ Tem cells and CLP scores (Figure 6F).

### 2.7. Association of CD24-MYB with Immature B-Cell Programs

Under physiological conditions, pro-/pre-B cells are largely restricted to the bone marrow. *IGH*-associated blood transcript changes in the peripheral blood of SACC patients, along with an inferred pro-/pre-B cells enrichment in lung metastases motivate a working hypothesis of systemic disruption of hematopoiesis in these individuals. In one index patient, two tumor-matched *MYB-NFIB* transcript junctions were detected in plasma cell-free RNA, and the higher-abundance junction was also detected in RNA from the cellular blood fraction (Supplemental Figure S1), positive RNA amplicons were confirmed by Sanger sequencing. Although *MYB* and *NOTCH1* were not identified as DEGs in peripheral blood, it remains plausible that circulating tumor-derived factors may remotely modulate *MYB/NOTCH1* signaling within the bone marrow niche, an effect that would not be reflected in peripheral blood transcriptomes. Nevertheless, direct mechanistic validation remains constrained, as ethical considerations preclude bone marrow aspiration in SACC patients, necessitating the use of public blood and bone marrow datasets in the present study.

**Figure 7:**
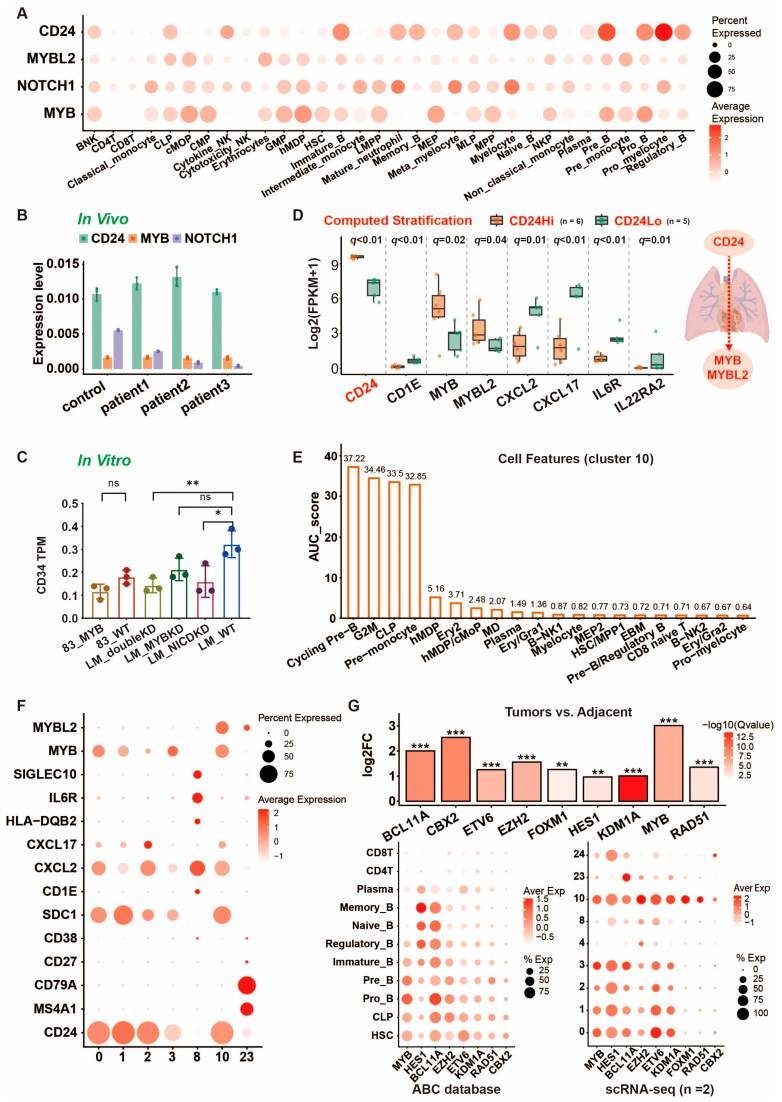
Candidate associations between ACC driver genes and bone marrow defects. (**A**) Expression of *CD24*, *MYB*, *MYBL2*, and *NOTCH1* across a spectrum of 32 bone marrow and blood cell types from the ABC database. (**B**) *CD24*, *MYB*, and *NOTCH1* are highly expressed in blood from both healthy controls and patients, as validated by qPCR. (**C**) *MYB* knockdown reduces *CD34* expression in SACC-LM cells, as assessed by RNA-seq, Mann-Whitney U test, *p* > 0.05 (ns), *p* < 0.05 (*), *p* < 0.01 (**). (**D**) Genes associated with *CD24* by computational stratification. (**E**) Based on AUC scores from the ABC database, cluster 10 is transcriptionally most similar to cycling pre-B, G2M, CLP, and pre-monocyte cells. (**F**) Literature-based hypothesis linking tumor-cell *CD24* to macrophage SIGLEC10. Clusters 0–3,10: cancer cells; 10: CSCs; 8: macrophages; 23: B cells. (**G**) Overlap between genes expressed in cluster 10 and HSC reference signatures, which is elevated in primary and metastatic ACC, only the log_2_FC and *q* values from the differential expression analysis of primary tumors are displayed (upper). *p* < 0.01 (**), *p* < 0.001 (***).

Publicly available ABC data profile the expression of both *NOTCH1* and *MYB* across various HSPCs (HSC, MPP, CMP, GMP, LMPP, MLP, hMDP, etc.). *MYB,* however, exhibits a wider expression range, encompassing pro-B, pre-B, and immature B cells. Furthermore, *MYBL2* expression is more concentrated in pre-B and immature B cells, strongly suggesting a critical role for *MYB/MYBL2* in B-cell development. Additionally, *CD24,* which was aberrantly expressed in lung metastases, is a canonical B cell surface marker that is broadly expressed on pro-B, pre-B, immature, naïve, and memory B cells (Figure 7A). qPCR validation confirmed *MYB*, *NOTCH1*, and *CD24* expression in PB (Figure 7B). Subsequent RNA sequencing demonstrated that *CD34* was significantly downregulated in SACC-LM cells upon dual knockdown of *MYB* and *NOTCH1* (Figure 7C), suggesting an association between *MYB* and the progenitor B-cell-like transcriptional state.

In metastatic-tissue expression strata, higher *CD24* expression was associated with higher of *MYB* and *MYBL2*, and lower expression of *CD1E*, *CXCL2*, *CXCL17*, *HLA-DQB2*, *IL6R*, and *IL22RA2*. Computational analysis suggested a potential association between the *CD24–MYB/MYBL2* axis and an immature, progenitor-like B-cell state (Figure 7D).

Marker genes of cluster 10 overlapped with cycling pre-B, G2M, common lymphoid progenitor, and pre-monocyte reference states (Figure 7E). Among these, shared genes including *MKI67*, *CCNB1/CCNB2*, *CDK1*, and *TOP2A* were enriched for proliferation-associated functions, leading to the designation of cluster 10 as a cell-cycle-high tumor-cell cluster, with signature genes also including *CD24*, *MYB*, *MYBL2* and *SDC1 (CD138)* (Figure 7F, Supplemental Table S6).

In the single-cell dataset, *CD24* was expressed in B cells and in tumor-cell cluster 10 (Figure 7F), which promotes macrophage ‘don’t-eat-me’ signaling via SIGLEC-10 [32], illustrating one mechanism by which cluster 10 cells evade immune attack.

Cell-level expression mapping further revealed overlapping expression of *MYB* with selected hematopoietic, epigenetic, cell-cycle, and DNA-repair genes, including *HES1*, *BCL11A*, *EZH2*, *ETV6*, *KDM1A*, *RAD51*, *CBX2*, and *FOXM1*, both in the HSC population of the ABC dataset and in cluster 10 of the single-cell datasets from two patients. (Figure 7G). Notably, these genes were consistently highly expressed in both primary (Figure 7G) and metastatic tumor tissues. Furthermore, the transcriptional regulatory trajectories of cluster 10 cells resembled those of HSCs, but were more consistent with a B-cell-like transcriptional signature.

## 3. Disscussion

Computational analyses suggest dysregulated HSPC differentiation in the immunocold phenotype of SACC, supported by prior detection of myeloid-derived suppressor cells (MDSCs), naïve B and double-negative T cells (DNTs) in primary tumor specimens [33] and further delineated by a spatial-temporal regulatory network of immune aberrancy. However, definitive validation requires experimental confirmation in patient bone marrow and physiologically relevant models.

First, transcriptomic deconvolution of peripheral blood revealed the concomitant presentation of hypolymphocytosis, developmentally aberrant BCR repertoires, myeloid activation, and index-case PCR evidence of circulating *MYB-NFIB* fusion, predicting hematopoietic dysregulation. PCR-confirmed upregulation of plasma *IL-33* and *CCL14* further corroborates this dysregulation, also pointing to a non-canonical, T cell-independent type II inflammatory state consistent with prior reports [34]. Deconvolution analysis indicated defective antigen-presenting cells (APCs) commitment, evidenced by hMDP accumulation in primary tumors, downregulation of *IL3RA* (expressed in LMPP/GMP/hMDP/cMOP) and *IL5RA* (plasma cells), and broad *HLA-I/II/III* downregulation [35]. Although monocytes differentiate into moDCs through *CX3CL1–CX3CR1* crosstalk at primary and metastatic sites, these moDCs—unlike conventional cDCs—primarily activate memory and TRM cells, driving Th1/Th17 responses [36,37]. Single-cell analyses confirm resident T cells and associated Th1/Th17 gene signatures in tumor tissues (Figure 6E). Conversely, lung metastases exhibited a significant increase in immature B cells that failed to activate resident or memory T cells via HLA class II presentation [38]. This finding is supported by the presence of CD8 naive, CD4 Tcm, and Th1 cells in lung metastases. We therefore propose that SACC immune coldness may primarily stem from defective APC differentiation, whereas ineffective immune inflammation in metastases results from impaired B cell–mediated antigen presentation.

Second, based on a virtual cell knockdown model, a signaling molecule regulatory cascade model was computationally derived: (1) primary site: The *IL33→PDGFRB/PDGFD→PDGFA/B→IL17B→IL17RB→OLIGI→NOTCH1* axis suppresses tumor cell *HLA-I* expression. Single-cell profiling revealed *NOTCH1* and *IL17B* expression in tumor cells and hMDPs [33]. This expression pattern suggests that either *NOTCH1* signaling or the *IL17B→IGF2* axis inhibits *HLA-II* expression in hMDPs, thereby impairing APC maturation and antigen presentation; (2) metastatic site: *CD24/IL17RB→MYB/MYBL2→NOTCH1→CXCL13/CXCR5* signaling restricts B cell maturation, potentially serving as a determinant of immune exclusion. The pubic ABC dataset confirmed co-expression of *CD24*, *MYB*, and *MYBL2* in immature B cells, pre-B cells, or hMDP cells, while *NOTCH1* and *MYB* co-localized in progenitor cells like HSCs and LMPPs, supporting the possibility that MYB or NOTCH1 abnormalities in HSCs contribute to immune cell misdifferentiation [39,40]. Thus, NOTCH1 may contribute to the immune desert phenotype. To date, *NOTCH1* inhibitors have not demonstrated clinical efficacy, largely owing to unacceptable toxicity profiles.

Third, Lung metastasis samples with low co-expression of *NOTCH1* and *MYB* were rarely observed [41]. Furthermore, stratified analysis was limited by uniformly high *TP53* expression. Notably, *NOTCH1*, *MYB*, and *TP53* were all highly expressed in both tumor cells and hMDP, suggesting they may mediate lung metastasis by altering hMDP differentiation [42]. Transcriptionally, we identified *TP53* as a major upstream regulator, activating *FOXP4* to enhance *IL17RB* expression and thereby amplifying *NOTCH1* signaling. *TP53* mutations may upregulate or disrupt *KMT6A* (*EZH2*) and *KDM5D*, contributing to chromosomal instability and tumor progression [43,44,45]. In addition, tumor cells exploit *PDGFRA* mutations to convert *IL-33* into an oncogenic cytokine. These findings nominate IL17RB as a candidate convergent therapeutic target in both primary and metastatic immune evasion programs, and preclinical efforts to target *IL17RB* are ongoing [30,46]. However, SACC-specific validation, including protein confirmation, target-specific perturbation, and in vivo efficacy and safety testing, is still required.

Finally, cluster 10 identified earlier exhibited marker genes closely aligned with both HSCs and progenitor B cells. This dual similarity suggests a potential role in impairing normal CLP stem cell differentiation [47]. Additionally, while no viral integration was detected across five tested viruses in sequenced samples (Supplemental Table S7), the origins of the *MYB* fusion and *NOTCH1* mutation remain unclear. Although somatic hypermutation (SHM) is typically confined to BCR recombination in germinal centers of the lymph node, aberrant differentiation of bone marrow stem cells—which upregulates *NOTCH1* and *MYB*—may create a permissive context for SHM-like mutagenesis, warranting experimental validation [48,49].

## 4. Limitations

Several limitations warrant careful consideration when interpreting the findings of this study. First, the peripheral blood cohort was modest in size and intended primarily for discovery; we therefore adopted permissive thresholds (|Log_2_FC| > 1, raw *p* < 0.05) to maximize detection sensitivity, which inevitably elevates the false-positive rate and limits the generalizability of the resulting gene list. Second, the single-cell dataset was derived from only two independent donors, and the primary and metastatic specimens were not patient-matched, precluding within-patient paired comparisons. This restricted sample size also renders the dataset underpowered for population-level inference, pseudotime trajectory analysis, and quantitative assessments. Third, bone marrow specimens from SACC patients were not available; the ABC reference atlas used for comparison represents healthy blood and bone marrow rather than a disease-matched comparator, which may obscure malignancy-specific alterations. Fourth, the inferred molecular regulatory networks rest entirely on bioinformatic predictions and await experimental validation in appropriate model systems, particularly to resolve their spatial architecture. Fifth, circulating *MYB-NFIB* fusion was assessed in a single index patient; given the scarcity of suitable specimens, this preliminary observation requires confirmation in larger, independent cohorts. Finally, critical covariates, including tumor burden, timing of metastasis, inflammatory comorbidities, and other sources of clinical heterogeneity, were not systematically captured, and the sample size precluded reliable multivariable adjustment. Residual confounding therefore cannot be excluded, and the associations reported here should be interpreted as hypothesis-generating rather than conclusive.

## 5. Materials and Methods

### 5.1. Patient Data

Peripheral blood samples from 22 SACC patients and 9 healthy controls were prospectively collected between 2024 and 2025, with blood PCR performed on one patient sample as a confirmatory assay. Patient characteristics are detailed in Supplemental Table S8. We additionally integrated published transcriptomic data (GSE282732) comprising: 25 primary tumors and 12 adjacent normal tissues; 37 lung metastatic tumors and 19 adjacent normal lung tissues [50]. All were processed with identical experimental protocols. Comprehensive patient characteristics are detailed in Supplemental Table S7, excluding those with preoperative anti-tumor therapies (e.g., radiotherapy/chemotherapy) or major immune system disorders.

### 5.2. RNA Extraction and qRT-PCR

Total RNA was isolated from fresh-frozen samples using E.Z.N.A. Total RNA Kit I (Omega Bio-Tek, Norcross, GA, USA). cDNA synthesis employed PrimeScript RT Kit (Takara, Kusatsu, Shiga, Japan) under standard cycling: 37 °C/15 min → 85 °C/5 s. qPCR was performed with SYBR Premix Ex Taq (Takara, Kusatsu, Shiga, Japan) using: 95 °C/30 s initial denaturation; 40 cycles of 95 °C/5 s → 60 °C/30 s. GAPDH- and actin-normalized relative expression was calculated via 2^−ΔΔCt^. Given that the Ct values of healthy controls exceeded 35 or were undetected, we normalized the 2^−ΔΔCt^ values of the normal group to 0. Primer sequences are in Supplemental Table S9.

### 5.3. Bulk Transcriptome Sequencing

This study utilized standardized RNA-seq protocols: Peripheral blood from 21 SACC patients and 9 healthy controls (PAXgene tubes, Becton, Dickinson and Company, Franklin Lakes, NJ, USA, Agilent RIN > 7) underwent Illumina NovaSeq X Plus sequencing at Xinanlou Biotech. Tissue data derived from our published cohort (GSE282732; n = 89 cases) and 4 additional tumor/adjacent samples were processed identically via MGISEQ2000RS (BGI) with TRIzol extraction (RIN ≥ 7). All data were uniformly analyzed using HISAT2 (v2.2.1, RRID:SCR_015530) alignment to GRCh38 (ENSEMBL r112, RRID:SCR_002344), StringTie (v2.1.6, RRID:SCR_016323) quantification, and FPKM normalization. Notably, initial differential expression analysis of blood samples (n = 30, ~35,000 genes) under stringent thresholds (|log_2_FC| ≥ 1, adjusted *p*-value < 0.05) yielded only 32 significant DEGs. Given the dual constraints of limited sample size and high data dimensionality, we implemented a discovery-phase approach using relaxed statistical criteria (|log_2_FC| > 1, *p*-value < 0.05) to mitigate excessive filtering of biological signals. This strategy yielded 100 candidate DEGs. Critically, qPCR validation confirmed concordant expression trends for selected genes between sequencing data and experimental results.

### 5.4. Pan-Cancer Analysis and Survival

Using TCGA (RRID:SCR_003193) data, we assessed *IL33* differential expression across 33 human cancers. Statistical significance (Wilcoxon test) is denoted: * p < 0.05; ** p < 0.01; *** *p* < 0.001. Overall survival was evaluated by Kaplan-Meier analysis with time-to-event in months, implemented via R (v4.4.3, RRID:SCR_001905) packages survival (RRID:SCR_021137) and survminer (RRID:SCR_021094).

### 5.5. Single-Cell Transcriptomics Analysis

scRNA-seq data from our published cohort (GSE216852) were reanalyzed, including one primary SACC and one lung metastasis case (3′-end sequencing) [30]. Peripheral blood data were integrated from public repositories [26,51]. All data were processed through a unified Seurat workflow: quality control (mitochondrial content <20%), log-normalization, PCA-based dimensionality reduction, graph-based clustering (resolution = 0.5). Cluster 4 was partitioned into 7 distinct subsets after standardization (resolution = 0.3), followed by pseudotime analysis using monocle3 package (RRID:SCR_018685).

### 5.6. Gene Knockdown and Sequencing In Vitro

As reported previously [30], stable *NOTCH1*-KD and *NOTCH1/MYB* double-KD cell lines were generated in SACC-LM/SACC-83 cells (RRID:CVCL_H590, RRID:CVCL_H589) using lentiviral shRNAs in pLKO.1-Puro or pCDHO-Neo-CMV-3-Flag vectors, with selection using 2 μg/mL puromycin or 1 μg/mL neomycin, respectively. Bulk RNA-seq data from these established lines are publicly available under GEO accession GSE216852. In the present study, additional RNA-seq data from NOTCH1-KD SACC-83 cell lines were newly deposited, generated using the same experimental and analytical procedures as previously described [30].

### 5.7. Immune Cell Definition and Quantification

We profiled immune cell enrichment using RNA-seq data from three biological compartments: PB, primary tumors, and lung metastases. A curated panel of 75 immune cell subtypes spanning seven lineages (progenitor cells, myeloid cells, Erythroid-Megakaryocyte progenitors, B cells, CD8^+^ T cells, CD4^+^ T cells, NK cells) was analyzed using integrated marker genes from ImmuCell AI (RRID:SCR_027645), XCell (RRID:SCR_026446), and UCSC (RRID:SCR_005780) databases (Supplemental Table S10).

ssGSEA (RRID:SCR_026610) was implemented via a custom Python 3.9 (RRID: SCR_008394) pipeline to quantify cell type abundance in individual samples. This method ranks genes by expression magnitude and computes a weighted cumulative enrichment score for predefined gene sets, prioritizing highly expressed markers using an exponential decay weighting factor (α = 0.25) [28]. Scores were normalized by the maximum absolute deviation across gene rankings to ensure cross-sample comparability. Expression matrices underwent log_2_ (FPKM + 1) transformation prior to analysis, with cell types exhibiting <10 overlapping marker genes excluded. Differential abundance between tumor samples and normal controls was assessed independently per cell type and biological compartment using the non-parametric Mann-Whitney *U* test (two-tailed; α = 0.05) after confirming non-normally distributed scores (Shapiro-Wilk test, *p* < 0.05). P-values were adjusted for multiple comparisons across 75 cell types and three compartments via Benjamini-Hochberg FDR correction (significance threshold: *q*< 0.05). Critically, differential cell types were defined only when demonstrating significant enrichment (*p* < 0.05) in both XCell and ImmuCellAI databases concurrently with consensus positivity in our in-house enrichment analysis.

### 5.8. RNA-seq Data Stratification and Correlation in Python

The stratification and the construction of the regulatory networks were implemented in Python 3.10 using a custom computational framework (Supplemental Table S11). Gene expression values were stratified into high/medium/low tiers based on expression quantiles, with differential analysis between high and low tiers performed using the core logical framework of DESeq2 (RRID:SCR_015687). Regulatory networks were constructed via GENIE3 (RRID:SCR_000217) to predict upstream/downstream molecular interactions, followed by report generation of hub genes or specific genes (code and dependent packages can be found in GitHub, https://github.com/acc837/stratified-difference-analysis, accessed on 18 July 2026; RRID:SCR_002630). For transcription factor co-expression profiling, Spearman correlation coefficients (ρ) were computed between each transcription factor and a target oncogene across matched tumor and adjacent tissue datasets. Differential correlation analysis was conducted using Fisher’s z-transformation with Benjamini-Hochberg FDR correction (significance threshold: *q* < 0.05). Tumor-enhanced transcriptional regulators were identified based on: (1) statistically significant difference in malignant versus normal tissue (FDR-adjusted *p* < 0.05), and (2) absolute correlation magnitude exceeding normal tissue levels ($\left|\rho_{tumor}\right|$ > $\left|\rho_{adjacent}\right|$). Prioritized candidates were ranked by minimal FDR and maximal absolute correlation difference (|Δρ|). Identical analytical methodology was extended to additional key pathway components.

### 5.9. Assay for the MYB-NFIB Fusion Gene in Blood

Tumor RNA was isolated using TRIzol (Invitrogen, Carlsbad, CA, USA). Libraries were prepared via the NEBNext Ultra II RNA Kit (NEB, Ipswich, MA, USA) and a customized 277-gene Agilent SureSelectXT RNA panel. Sequencing was performed on an MGI DNBSEQ-T7 platform (RRID:SCR_024847). Reads were aligned to hg19 using BWA (v0.7.15; BWA-MEM). Gene fusions were identified by STAR-Fusion (v1.8.1; RRID:SCR_025853) and validated using the Integrative Genomics Viewer (IGV v2.8.0; RRID:SCR_011793).

Plasma and blood cell RNA were extracted using Starvio cfRNA (Starbio, Shanghai, China) and Magen RNA (Magen Biotech, Guangzhou, China) kits, respectively. Nested PCR primers were designed to span the gene fusion junction, with the outer and inner primer pairs (Supplemental Table S8). Fusion transcripts were amplified by two-round nested-PCR using 2× Hieff^®^ PCR Master Mix Kit (YEASEN Biotechnology, Shanghai, China) and SLAN-96S Real-Time PCR System (RRID:SCR_027087). Cycling conditions were: 98 °C for 3 min; 35 cycles of 98 °C (10 s), 60 °C (30 s), and 72 °C (30 s); and a final 10-min extension at 72 °C. Amplicons were verified via Sanger sequencing (ABI 3500Dx) and analyzed using Chromas (RRID:SCR_000598).

### 5.10. Statistical Analysis and Visualization

Differential gene expression analysis was performed using the DESeq2 package via the Dr. Tom multi-omics data mining system (https://biosys.bgi.com, RRID:SCR_027646). Intergroup comparisons were conducted using the non-parametric Mann-Whitney *U* test. All gene expression heatmaps, dot plots, box plots, violin plots, t-SNE projections, and UMAP visualizations were generated using R (v4.4.3, with ggplot2; RRID:SCR_014601, and Seurat packages; RRID:SCR_016341). Gene expression plots for qPCR validation were created using GraphPad Prism (v10.1.2; RRID:SCR_002798). Statistical significance for DESeq2 analyses were defined as follows: *q* < 0.05 (*), *q* < 0.01 (**), *q* < 0.001 (***), *q* < 0.0001 (****). In this study, the term “q value” refers to the Benjamini–Hochberg-adjusted P value returned by DESeq2. ALL schematic elements were obtained from BioRender (RRID:SCR_018361).

## 6. Conclusions

In summary, *NOTCH1* governs T-cell/dendritic cell maturation while *MYB* drives B-cell differentiation, though these regulatory roles remain computationally inferred and require experimental validation. Clinical translation will require validation in larger independent cohorts, confirmation at the protein level, patient-derived perturbation models, and in vivo efficacy and safety studies.

## Data Availability

The sequencing data generated in this study (including PB, tissues, and cell lines) have been deposited in the Genome Sequence Archive under accession number HRA017018. Upon publication, researchers may access the data upon reasonable request and with permission from the corresponding author.

## Supplementary Materials

The following supporting information can be downloaded at: https://www.mdpi.com/article/10.3390/ijms27146498/s1.

## Abbreviations

ABC	Atlas of human blood cells
APCs	Antigen-presenting cells
Baso	Basophil
BCR	B-cell receptor
cDCs	Conventional dendritic cells
CLP	Common lymphoid progenitor
CMP	Common myeloid progenitor
CSCs	Cancer stem cells
DC	Dendritic cell
DEGs	Differentially expressed genes
DNTs	double-negative T cells
Eos	Eosinophil
Ery	Erythrocyte
GMPs	Granulocyte-macrophage progenitors
HLA	Human leukocyte antigen
hMDP	Human myeloid dendritic progenitor cells
HSC	Hematopoietic stem cell
HSPCs	Hematopoietic stem/progenitor cells
ICI	Immune checkpoint inhibitors
iDCs	Immature dendritic cells
iNKT	invariant natural killer T cell
iTregs	Induced regulatory T cells
LDAs	Leukocyte differentiation antigens
Mac	Macrophage
MAIT	Mucosal-associated invariant T cells
MB	Myeloblast
MC	Mast cell
MDSCs	myeloid-derived suppressor cells
MHC	Major histocompatibility complex
MK	Megakaryocyte
moDCs	Monocyte-derived dendritic cells
Mono	Monocyte
MPPs	Multipotent progenitors
Neut	Neutrophil
PB	Peripheral blood
PC	Plasma cells
pDCs	Plasmacytoid dendritic cells
PLT	platelet
SACC	Salivary adenoid cystic carcinoma
SHM	Somatic hypermutation
SL	Small lymphocyte
Tcm	Central memory T cells
TMB	Tumor mutation burden
TRM	Tissue-resident memory T-cells
VECs	Vascular endothelial cells
ssGSEA	Single-sample gene set enrichment analysis
