# Supplementary material for: Integrated Transcriptomic Analysis of NOTCH1- and MYB-Associated Immune Features in SACC
Source: Int J Mol Sci. 2026 Jul 22;27(14):6498. doi: 10.3390/ijms27146498 (PMC13410102; doi:10.3390/ijms27146498)
Supplement: Supplementary file 1 [file ijms-27-06498-s001.zip › supplemental figures.pdf]

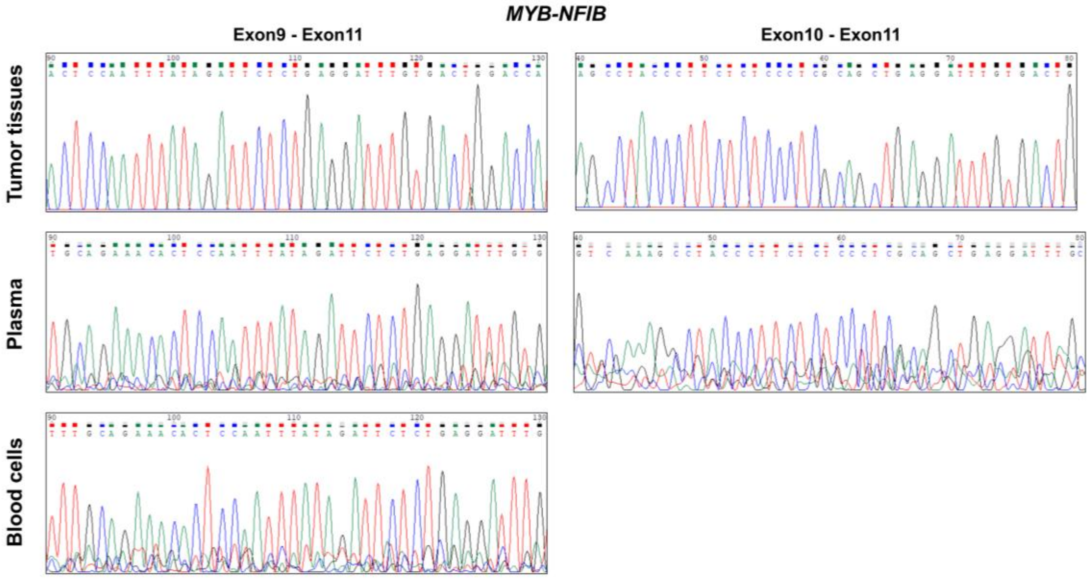

Supplemental Figure S1. Tumor-derived *MYB-NFIB* fusion gene detection in peripheral blood. RNA samples extracted from tumor tissue and peripheral blood were analyzed by nested PCR followed by Sanger sequencing. Two fusion variants were identified in patient-matched primary tumor tissue: MYB exon 9–NFIB exon 11 and MYB exon 10–NFIB exon 11. Both fusion forms were detected in RNA from cell-free plasma, whereas only one variant was detected in RNA isolated from whole blood cells.

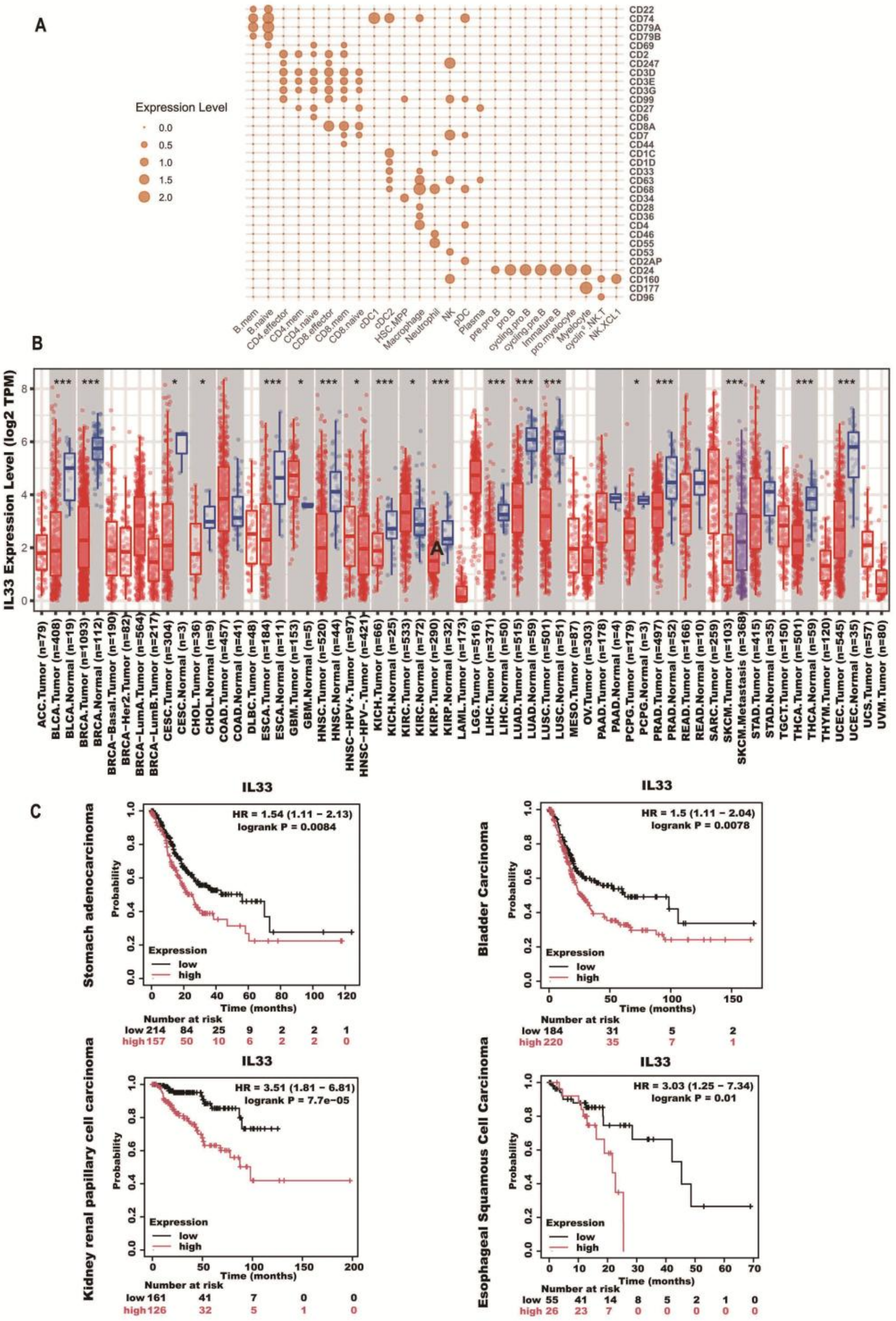

Supplemental Figure S2. Validation of LDA and IL33 expression and their association with clinical prognosis based on public databases. **A)** Differentially expressed LDAs in cancer tissues were mapped to their source immune cell types via reference-guided annotation of blood scRNA-seq ABC profiles. **B)** Pan-cancer analysis of *IL33* based on TCGA database. Blue indicates normal tissues, and red indicates tumor tissues. **C)** Association between high *IL33* expression and poor overall survival across multiple human

tumors.

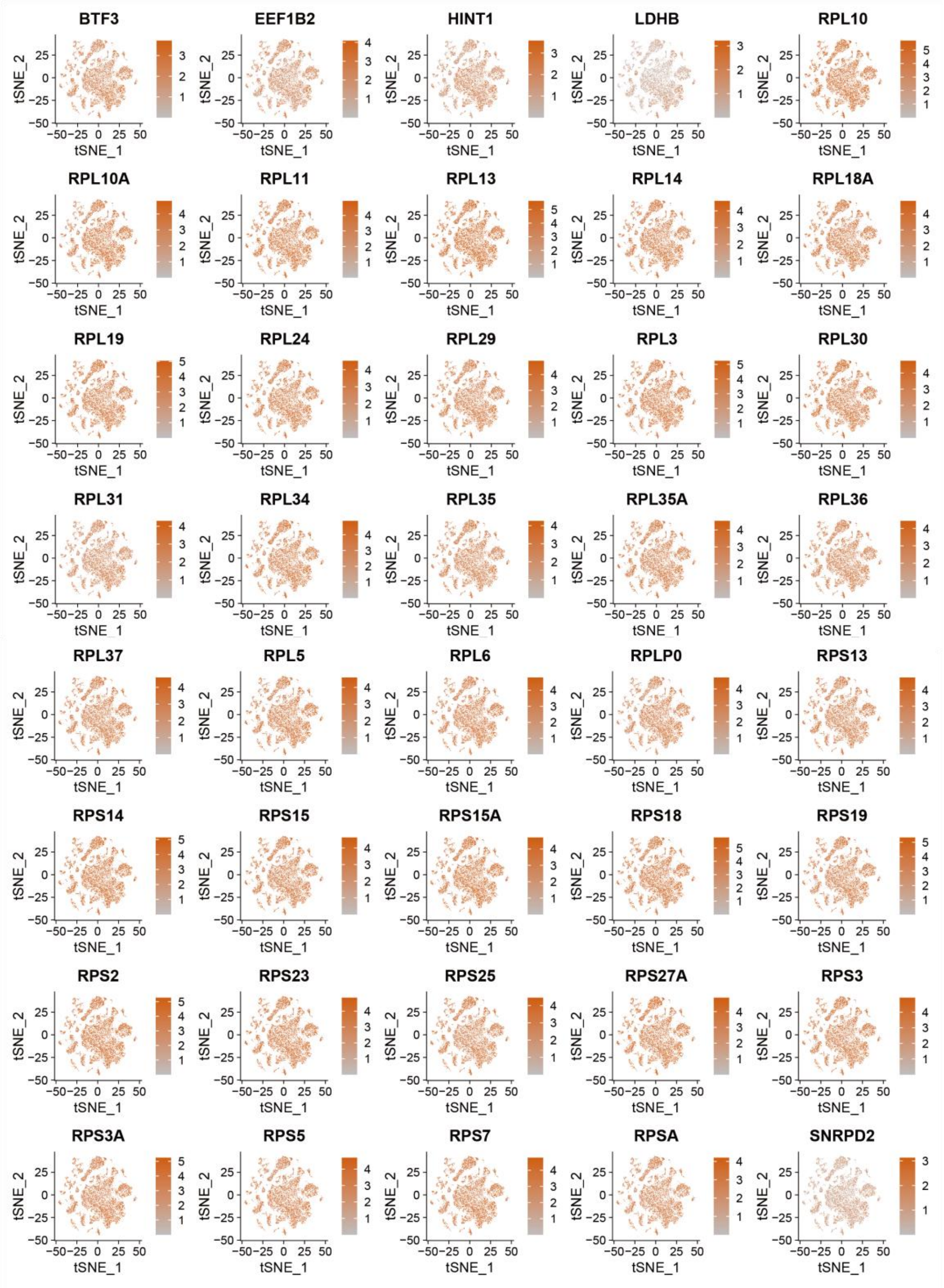

Supplemental Figure S3. t-SNE plot visualizing the expression patterns of the top 50 marker genes for hMDP.
